# A Review of the Enablers and Barriers of Medical Student Participation in Research

**DOI:** 10.1007/s40670-024-02156-z

**Published:** 2024-09-04

**Authors:** Chance Mayne, Hannah Bates, Devang Desai, Priya Martin

**Affiliations:** 1 Department of General Medicine, Toowoomba Base Hospital, Toowoomba, QLD Australia; 2 Department of Internal Medicine, Royal Brisbane and Women’s Hospital, Herston, QLD Australia; 3 Director of Urology, Toowoomba Hospital, The University of Queensland, Brisbane, Australia; 4 Occupational Therapy, School of Health and Medical Sciences, University of Southern Queensland, Toowoomba, QLD 4350 Australia; 5 Rural Clinical School, Faculty of Medicine, The University of Queensland, Locked Bag 9009, Toowoomba, QLD 4350 Australia

**Keywords:** Medical student, Student research, Extracurricular, Enablers, Barriers

## Abstract

Although research is an integral component in medicine, student participation in research remains limited. This is a systematic review conducted using rapid review methods conforming to the WHO and Cochrane guidelines to synthesise evidence on the enablers and barriers of medical student participation in research. PubMed, EMBASE, and PsycINFO were searched, yielding 27 final studies. Most studies were single-centre studies, and all were cross-sectional. All were quantitative except for one mixed-methods study. Identified barriers and enablers were mapped onto the micro, meso, and macro frameworks. There are more perceived barriers than enablers of medical students’ participation in research. The micro, meso, and macro frameworks provide a useful system to unpack and tackle the barriers.

**Supplementary Information:**

The online version contains supplementary material available at 10.1007/s40670-024-02156-z.

## Introduction

Research is an integral component of evidence-based medicine. Relevant and timely information about novel disease management, pharmacological advancements, and public health interventions is critical in modern healthcare. In this context, research has become a considered method of skill development and literature interpretation for clinicians [1]. In addition to this, medical research has a direct impact on the relationship between patients and the healthcare system, and their trust in ever-evolving medical practices [2]. Engaging pre-qualification/registration (i.e. primary degree to obtain registration as a doctor) medical students in research from their training period could be a useful strategy to facilitate capacity building and a positive research culture right from the start.

Combining clinical work and research is no easy feat. For example, the proportion of physicians engaged in research in the United States dropped from 4.7% in the 1980s to approximately 1.5% in 2019 [3]. These statistics partly reflect the increasing demands and workload of clinical practice, the financial costs of pursuing both clinical practice and research, and the specialisation and knowledge required for undertaking research [4]. It has been shown that physicians who participated in research during their time in medical school are more likely to contribute to greater research later in their careers [5]. Despite such evidence, medical student research participation rates remain low [6, 7].

Although medical students view research opportunities as catalysts to obtaining entry into specialty training pathways, their participation in research remains limited [8]. There is minimal research requirement as part of speciality training, and it depends on the interest of the individual along with the institution as to whether the individual is involved in research activities. Existing literature reveals several barriers that hinder medical student research participation including lack of opportunities, difficulty finding suitable supervisors and mentors, and a lack of time [6, 7]. Whilst the benefits of research involvement in medical school are favoured, student perception of research in this phase appears to be less favoured [9]. The benefits of students participating in research do not appear to translate to higher levels of participation [10]. A comprehensive synthesis on the current evidence related to enablers and barriers of medical student participation in research is required to inform policy and practice to mitigate these issues.

Previous research has utilised the micro, meso, and macro frameworks to unpack enablers and barriers of an investigated topic [11, 12]. Macro factors involve policy considerations including legal, regulatory, and economic factors. Meso factors are related to the organisation and community, and micro factors are at the team and individual levels in the context of day-to-day practice. This review will utilise this framework to systematically unpack the enablers and barriers of medical student research participation at these levels. Doing so will not only enhance translation of review findings to policy and practice, but it will also strengthen the scholarship in this area. A preliminary mapping exercise of enablers and barriers to the macro, meso, and micro levels, based on information from the preliminary scoping searches, was completed during protocol development [13]. The aim of this review was to synthesise the evidence on the enablers and barriers of research participation among students undertaking their pre-qualification medical studies, building on existing conceptual frameworks.

## Methods

Whilst using rapid review methodology considering available resources to undertake the review, systematic search methods were employed to ensure rigour. The review followed the World Health Organization (WHO) and the Cochrane guidelines for the conduct of rapid reviews [14, 15]. A protocol was developed and registered on the Open Science Framework [13]. The WHO checklist for rapid reviews to demonstrate quality assurance of the review can be found in Supplementary Table 1.

### Search Strategy and Data Sources

The databases searched in this review included PubMed, EMBASE, and PsycINFO. This decision was made following a preliminary scoping search to identify sources with the most relevant citations of the review topic. Detailed PICo (Population, Investigated phenomena and Context; Table 1) domains were used to create inclusion and exclusion criteria. Essentially, quantitative, qualitative, and mixed-methods studies of medical students investigating the enablers and barriers to research participation during their medical school years, within university and healthcare settings, were included. Supplementary Table 2 contains search strategies for all three included databases. Searches were run in July 2022 and updated in October 2023.

**Table 1:** Inclusion and exclusion criteria

	Inclusion criteria	Exclusion criteria
Population	Medical students (both undergraduate and postgraduate entry)	Research masters (i.e. M.Phil) or PhD students, non-medical students (i.e. nursing, allied health, etc.), provisional entry students (i.e. pre-medical degree)
Investigated phenomena	Barriers and/or enablers to participation in research during medical school	Participation in activities other than research (e.g. other work experience, teaching, training)
Context	Research in medical school or health settings while on placement	Research outside of these contexts
Study design	Primary research studies -Quantitative designs (including RCTs, cohort studies, pre-post, cross-sectional etc.) -Qualitative designs (including interviews, focus groups, case studies) -Mixed-methods designs	Secondary research (systematic reviews, other reviews) Editorials, opinion pieces, commentaries Position papers Conference abstracts and posters Research protocols
Other	English-language literature Publications dated 2012 onwards Full texts	Non-English literature Non-published studies (e.g. grey literature, thesis and dissertation manuscripts)

### Search Outcomes

All citations retrieved from the search were imported into Endnote X9™ [16] and de-duplicated. Screening of titles and abstracts against the inclusion criteria was conducted using Covidence™ [17]. For the title and abstract screening stage, 50 articles were screened by three reviewers (CM, HB, PM) together as a pilot exercise, with subsequent screening completed by two reviewers (CM, HB). Subsequently, at the full-text screening stage, three reviewers (CM, HB, PM) screened ten articles together to validate the process, with subsequent screening completed by two reviewers (CM, HB). During screening, conflict resolution was provided by a third reviewer (PM). Articles which met inclusion criteria were progressed to data extraction, while those which did not were excluded. The updated search did not yield any additional relevant articles.

## Methodological Quality

Methodological quality of included studies was assessed using the modified McMaster Quantitative Critical Appraisal tool [18] and the McGill Mixed Methods Appraisal Tool (MMAT) [19]. They were chosen as they are freely available and are widely used. Methodological quality was assessed by two reviewers (CM, HB) and verified by a third reviewer (PM). All discrepancies were resolved through mutual discussions by the review team. All studies, regardless of their methodological quality, underwent data extraction and synthesis.

### Data Extraction

A data extraction template (Supplementary Table 3) was developed and piloted on five studies by the review team prior to being finalised. Subsequent data extraction was carried out by two reviewers (CM, HB).

### Data Synthesis

A directed content analysis approach of a deductive nature was used to analyse the extracted data. Codes were developed using the micro, meso, and macro frameworks for both enablers and barriers. The data were then synthesised and mapped against these codes, and categories were developed for reporting [20]. This approach was chosen as it enables conceptual extension of theory to progress scholarship. Directed content analysis is a more structured process than traditional content analysis and can complement a structured review process [20]. Data synthesis was performed by three reviewers (CM, HB, PM) through regular discussions until consensus was reached to enable researcher-triangulation. The fourth reviewer (DD) validated the findings.

## Results

**Fig. 1:**
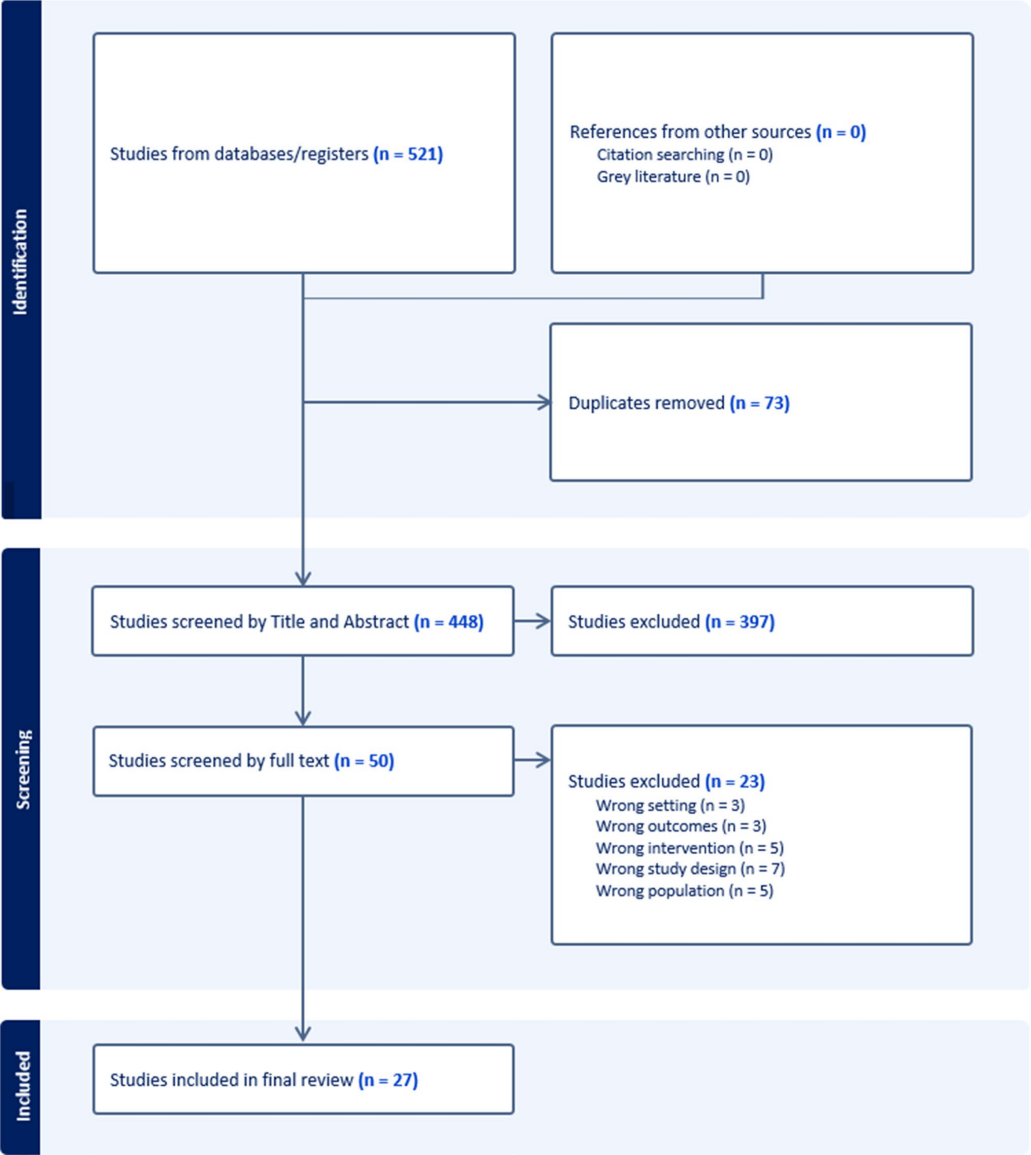
PRISMA flowchart of included studies

A total of 521 studies were extracted from the database search. Following removal of 73 duplicates, 448 articles were progressed to title and abstract screening. Subsequently, 50 studies were progressed for full-text screening. Of these, 23 studies were excluded based on wrong setting (*n* = 3), wrong outcomes (*n* = 3), wrong intervention (*n* = 5), wrong study design (*n* = 7), and wrong study population (*n* = 5), leaving 27 studies in the final review. A flow diagram of included studies is provided in Fig. 1. Further information on excluded studies with reasons is available in Supplementary Table 4.

Of the 27 included studies, eight originated from India, three each from New Zealand and Saudi Arabia, and four from Pakistan. One study each was from Australia, Colombia, Nigeria, Poland, South Africa, Sweden, and the United Arab Emirates. Lastly, one study was conducted across Egypt, Algeria, Sudan, Joran, Syria, and Palestine, while another was conducted across the UK, New Zealand, Malaysia, Canada, and France. The majority of studies (*n* = 22) were conducted in a university setting. The remaining studies were conducted in a hospital (*n* = 2), at a student conference (*n* = 2), or in a research programme setting (*n* = 1). Twenty-five studies surveyed only undergraduate students. One study surveyed undergraduate and intercalating medical students and one surveyed both undergraduate and post-graduate students. The sample size of the studies ranged from 48 to 2989 participants. The most widely used study design was a quantitative survey (*n* = 26), while only one study employed a mixed-methods design. There were no qualitative studies. Common variables measured across most studies included student demographic information, level of research knowledge, research practices/experiences (including publication rates), general attitudes towards research, enablers/motivators for research involvement, barriers to research involvement, and future research goals/intentions. Further information about study characteristics is available in Table 2.

**Table 2:** Study characteristics

Author	Country	Setting	Participants	Study design/measurement tool	Outcome measures
Alamri et al. 2019a	New Zealand	University	Undergraduate students (*n* = 48)	Mixed-methods: qualitative, cross-sectional survey + retrospective analysis	Publication rates, perceived barriers and motivators to pursuing research projects, supervisor satisfaction with research programme
Alamri et al. 2019b	New Zealand	University	Undergraduate students (years 2 and 3, *n* = 249)	Quantitative, cross-sectional survey	Research awareness and attitudes, motivators, and barriers to research
Alamri et al. 2021	New Zealand	University	Junior medical students (*n* = 253), intercalating medical students (*n* = 10), senior medical students (*n* = 323)	Quantitative, cross-sectional survey	Research engagement, motivation type (intrinsic vs extrinsic), effect of motivation type on other factors surrounding research engagement
AlGhamdi et al. 2014	Saudi Arabia	University	Years 4 and 5 undergraduate students (*n* = 172)	Quantitative, cross-sectional survey	Perceptions of research importance/impact; obstacles to research; research practice and experiences; sociodemographic information
Assar et al. 2018	Egypt, Algeria, Sudan, Jordan, Syria, Palestine	University	Undergraduate medical students (*n* = 2989)	Multi-centre, quantitative, cross-sectional survey	Demographics, knowledge, attitudes, practices, perceived barriers
Awofeso et al. 2020	Nigeria	University	Undergraduate medical students (*n* = 221)	Quantitative, cross-sectional survey	Knowledge of medical research, perceived barriers to research
Baig et al. 2013	Pakistan	University (private = 2, public = 2)	Medical students (*n* = 398)	Quantitative, cross-sectional survey	Year, institution, participation in research and intention, previous experience, satisfaction, incentives, suggestions for improvement
Bonilla-Escobar et al. 2017	Colombia	University	Medical students (*n* = 133)	Quantitative, cross-sectional survey	Sociodemographic information, research influences, affiliations, productivity, extracurricular activities, research motivations, career-research relations, research benefits and professional plans, personal attributes
Chellaiyan et al. 2019	India	University (private)	Undergraduate students (*n* = 344)	Cross-sectional survey	Demographic information, knowledge/perceptions of research, attitudes, practice, barriers
Funston et al. 2016	UK (*n* = 454) NZ (*n* = 115) Canada (*n* = 110) Malaysia (*n* = 106) France (*n* = 105)	University	Medical students		Demographic information, research perceptions (experience, barriers, financial implications), future goals (incentives, barriers, career goals)
Hada et al. 2021	India	Medical college, hospital	Undergraduate medical students (*n* = 292)	Cross-sectional survey	Demographic information, student attitude towards research, attitude for sustaining research interest (if reported involvement)
Jimmey et al. 2013	India	University, medical conference	Undergraduate (2nd–5th year MBBS, *n* = 114)	Cross-sectional survey	Research involvement, difficulties faced during research, reasons for research involvement
Kharraz et al. 2016	Saudi Arabia	University	Undergraduate students (years 2 and 3, *n* = 221)	Cross-sectional survey	Perceived barriers towards research engagement
Kini et al. 2017	India	University	Undergraduate students (*n* = 220)	Cross-sectional survey	Attitude towards research and its career impacts, perceptions towards research, obstacles to research
Kumar et al. 2019	Pakistan	University	Undergraduate (years 3–5,* n* = 687)	Cross-sectional survey	Barriers experienced by medical students in conducting research
Mahmood-Shah et al. 2017	Pakistan	University	Undergraduate (*n* = 294)	Cross-sectional survey	Attitudes, knowledge, and practices of research; perceived motivation, barriers, and interventions; intentions for future research
Muhandiramge et al. 2021	Australia	University	Undergraduate and postgraduate (*n* = 704)	Cross-sectional survey	Research experience; understanding of research terms; attitudes towards research (barriers, motivators)
Nel et al. 2014	South Africa	University	Undergraduate (*n* = 733)	Cross-sectional survey	Demographic information, interest in specialisation, extent of previous research involvement, general attitude towards research, factors influencing attitude towards research
Noorelahi et al. 2015	Saudi Arabia	University	Undergraduate (years 3–5, *n* = 233)	Cross-sectional survey	Attitudes to and practice of research, barriers to conducting research
Omprahash et al. 2019	India	University	Undergraduate (year 1, *n* = 205)	Cross-sectional survey	Knowledge of research, factors promoting positive attitude towards research and encouraging research involvement, perceived barriers to research
Rani and Priya 2014	India	University	Undergraduate 2nd + 3rd years (*n* = 308)	Cross-sectional survey	Demographic information, knowledge, and attitude of research methodology/statistics, research practices
Sayedalamin et al. 2018	UAE	Medical student conference	Undergraduate (*n* = 288)	Cross-sectional survey	Research attitudes and practices, barriers to research
Shahab et al. 2013	Pakistan	University	Undergraduate (year 2–5, *n* = 160)	Cross-sectional survey	Student knowledge of research activities, perceived barriers, student attitudes
Singh et al. 2021	India	Teaching hospital	Undergraduate (year 2, *n* = 217)	Cross-sectional survey (pre and post 2-year workshop)	Comparison of knowledge, attitude, and practice between pre and post survey
Sobczuk et al. 2022	Poland	University	Undergraduate (2nd year *n* = 391, 5th year *n* = 695)	Cross-sectional survey	Scientific interest and participation, opinions on research issues, perception of potential barriers to research
Stockfelt et al. 2016	Sweden	University	Undergraduate (*n* = 471)	Cross-sectional survey, mixed methods	Medical student interest in research, disincentives for research, how students get involved, types of research
Yerpude and Jogdand 2016	India	University	Undergraduate (final year, *n* = 282)	Cross-sectional survey	Demographic information; student knowledge, attitudes, and barriers

## Methodological Quality

A quality assessment was performed for each of the included studies, using the Modified McMaster’s Tool for quantitative studies (*n* = 26) and MMAT (*n* = 1) as outlined in Supplementary Tables 5a and b. All quantitative studies reported the study purpose and incorporated a relevant literature review. All studies were cross-sectional. All studies reported the sample size, but only 16 studies justified the sample size. Most outcome measures used were not reliable, and only under a quarter of studies used valid measures. A majority of studies (*n* = 20) reported results in terms of statistical significance. All but one study used appropriate analysis methods and all studies reported on clinical importance of findings. All but two studies presented appropriate conclusions. The different components of the mixed-methods study did not adhere to the quality criteria of each tradition of the methods involved but met all other criteria on the MMAT tool.

### Barriers and Enablers of Medical Student Participation in Research

Utilising the macro, meso, and micro frameworks, enablers and barriers identified were mapped to the corresponding levels. Enablers of medical student participation in research were mapped to macro (career and financial incentives) and micro (skill acquisition and interest in research) levels, with no identified enablers at the meso level. Barriers were identified at all three levels. At the macro level, lack of training/information and financial constraints were noted. At the meso level, studies described difficulty finding supervisors, and at the micro level a lack of time, interest, and impact on studies/training were highlighted.

#### Macro Level Enablers

Career and financial incentives were identified as enablers. Research was recognised in ten studies as an incentive to enter coveted medical training programmes and improvement of a doctor or student’s curriculum vitae [1, 5, 10, 21–27]. Financial incentives were identified in five studies as a motivator for student participation in research [10, 21, 23, 27, 28].

#### Micro Level Enablers

Skill acquisition and interest in research were the enablers identified at the micro level, interacting with day-to-day practice*.* Involvement in research for academic and skill development was the most recognised enabler of research participation in 11 studies [1, 5, 7, 10, 21, 24, 25, 27–29]. Personal interest in research or on a particular topic promoted student participation in research in ten studies [1, 5, 21–24, 26–28].

#### Macro Level Barriers

Lack of training or information and financial constraints were mapped to this level. The absence of formal research training or a general lack of research awareness in universities was associated with decreased knowledge of available research opportunities and skills to participate in 18 studies [2, 5, 7, 8, 10, 21, 25–27, 29–36]. Fourteen studies identified financial limitations as a barrier to research participation. Research was commonly completed on a volunteer basis or in conjunction with minimal financial aid through the enrolling university, which reduced student participation [1, 2, 5, 7, 21, 27–29, 31–36].

#### Meso Level Barriers

Highlighted by 11 individual studies, access to suitable research mentors was a significant hurdle to pursuing research as a student [1, 7, 10, 22, 23, 28, 31–33, 36, 37].

#### Micro Level Barriers

A lack of time, interest, and perceived impact on medical studies were barriers mapped to the micro level. Identified by 20 studies, increased workload and educational commitments related to medical studies limited time available to undertake research [1, 2, 5, 7, 10, 21–30, 32–36]. Ten studies identified a lack of interest in research as a barrier to participation during medical studies [8, 10, 21, 25, 26, 29, 31, 32, 35, 36]. Finally, participants in six studies noted that research participation in medical school might impact their medical education and prolong training [2, 5, 7, 21, 24].

## Discussion

A systematic review and meta-analysis of medical student research in 2015 highlighted the association between medical student participation in research and improved short- and long-term scientific productivity, more informed career choices, and long-term success in academia [38]. It further provided considerations for policy, decision makers, and researchers to progress this area. Despite these calls, engagement and participation of medical students in research and the resulting outputs and outcomes remain low [6]. Our review explored enablers and barriers to medical student participation in research in order to further understand the prevailing gap. We subsequently mapped the enablers and barriers onto a conceptual framework to unpack different layers involved. Barriers to participation appear to outweigh enablers, thus substantiating the low participation rates in the literature [6, 38]. While identified enablers were mapped only at two levels of the framework, namely macro and micro, identified barriers were mapped to all three levels, indicating the wider extent of barriers across the continuum.

The review by Amgad and colleagues exposed the lack of well-controlled high-quality prospective studies in this field [38]. Eight years later, our review too echoes this finding. Studies included in our review were predominantly cross-sectional in design and utilising surveys to investigate participant perspectives. Apart from one mixed-methods study, all studies were quantitative, with an absence of qualitative studies to unpack the ‘how’ and ‘why’. Further, the surveys used in the included studies did not have established psychometric properties. The risk with in-house developed surveys without established psychometric properties is well-known. The field can only move forward when robust measures and tools are available [39]. Given the diversity of approaches to research in different medical schools across the globe, availability and use of established surveys measures are essential. Furthermore, there is a need for qualitative studies so as to obtain in-depth experiences of students participating in research and their supervisors [6]. Without these in-depth perspectives, available evidence will remain restricted.

Medical students are a potential untapped resource that can be channelled to boost research outcomes. Medical students are generally interested and motivated to acquire new skills and education that enable them to progress their careers. However, some may find it hard to carry out research while juggling medical studies and associated workload. This could result in lower engagement and project completion rates. A recent Australian study by Fox and colleagues found that 33% of completed research projects medical students were involved in led to a peer-reviewed publication, while 51% led to outputs including conference presentations [6]. This is slightly higher than the rates reported in the systematic review and meta-analysis by Amgad and colleagues in which only 30% of medical student projects resulted in peer-reviewed publications. Unless barriers at all levels of the system are tackled, these rates are unlikely to improve [38].

Structured and targeted support that streamlines student participation and involvement in research from an early stage can make a difference. This can be enacted at several levels. At the macro level, institutions can provide medical students with a research framework, educational resources to enable research, mentorship, and supervision. Integrating research into the mandatory curriculum may be more facilitatory than undertaking research in an extracurricular capacity [38]. Provision of incentives and/or a good support and supervision structure could also assist students in not only engaging with research but also completing it to a high standard. At the meso and micro levels, research supervisors can improve research culture by providing adequate and high-quality supervision, and promoting and educating medical students on the outcomes of research, thus boosting interest and motivation. Further, early adoption of research, training, education, supervision, and culture is expected to ultimately improve medical student research participation.

Research may be highly sought after in some academic centres and countries that offer incentives to clinicians, researchers, and participants. However, in many other contexts such as within Australia, clinicians predominantly conduct research in their own time and are not financially incentivised. All academic titles are not remunerated and participation in research is voluntary. Institutions do not have a requirement for a certain number of research activities to be conducted; and hence, there is a large heterogeneity in the research output and quality amongst institutes. This highlights a systematic issue which needs to be addressed to promote research in all contexts.

### Strengths and Limitations

Most studies included within the review were single-centre studies involving one university. Several studies present a potential selection bias as students that completed surveys may have been the ones that were interested or involved in research. Several processes were used in this review to ensure rigour. This review, although using rapid methods, followed systematic searching and adhered to guidelines from the WHO and Cochrane for the conduct of rapid reviews. Use of the micro, meso, and macro frameworks has enabled the visualisation of more barriers than enablers in this field.

## Conclusion

There are more perceived barriers than enablers of medical students’ participation in research. These can be addressed at several levels including academic and healthcare institutions, research supervision and mentorship, financial incentives to students, and research and provision of a supportive and positive research culture. Academic and healthcare institutes can partner in several ways to provide more support, structure, and incentives for students to engage in research. Further studies are needed, especially using qualitative methods, to understand in-depth experiences of students and their supervisors engaging with research during the student’s medical training period.

## Supplementary Information

Below is the link to the electronic supplementary material.

Supplementary file1 (DOCX 40 KB)

## Data Availability

All available data from this review have been included in the main paper and supplementary information.

## References

[1] Alghamdi KM, Moussa NA, Alessa DS, Alothimeen N, Al-Saud AS. Perceptions, attitudes and practices toward research among senior medical students. Saudi Pharm J. 2014;22(2):113–7. 24648822 10.1016/j.jsps.2013.02.006 PMC3950504

[2] Assar A, Matar SG, Hasabo EA, Elsayed SM, Zaazouee MS, Hamdallah A, et al. Knowledge, attitudes, practices and perceived barriers towards research in undergraduate medical students of six Arab countries. BMC Med Educ. 2022;22(1):44. 35042492 10.1186/s12909-022-03121-3 PMC8767733

[3] Sobczuk P, Dziedziak J, Bierezowicz N, Kiziak M, Znajdek Z, Puchalska L, et al. Are medical students interested in research? - Students’ attitudes towards research. Ann Med. 2022;54(1):1538–47. 35616902 10.1080/07853890.2022.2076900 PMC9891220

[4] Laidlaw A, Aiton J, Struthers J, Guild S. Developing research skills in medical students: AMEE Guide No. 69. Med Teach. 2012;34(9):e754-71. 22905661 10.3109/0142159X.2012.704438

[5] Muhandiramge J, Vu T, Wallace MJ, Segelov E. The experiences, attitudes and understanding of research amongst medical students at an Australian medical school. BMC Med Educ. 2021;21(1):267. 33971858 10.1186/s12909-021-02713-9 PMC8108334

[6] Fox JL, Cribb J, Cumming K, Martin P. Medical student interest and participation in research at one rural clinical school: insights from the last six years. Aust J Rural Health. 2023;31(3):569–74. 36762881 10.1111/ajr.12970

[7] Funston G, Piper RJ, Connell C, Foden P, Young AM, O’Neill P. Medical student perceptions of research and research-orientated careers: an international questionnaire study. Med Teach. 2016;38(10):1041–8. 27008336 10.3109/0142159X.2016.1150981

[8] Shahab F, Ali MA, Hussain H. Involvement and barriers to research amongst students of Khyber Medical College. Journal of Postgraduate Medical Institute. 2013;27(3)

[9] Singh ABS, Beg MA, Kumar H. Evaluation of knowledge, attitude, and practice of undergraduate medical students toward medical research in a tertiary care teaching hospital. National Journal of Physiology, Pharmacy and Pharmacology. 2021;11(10):1125–9.

[10] Mahmood Shah SM, Sohail M, Ahmad KM, Imtiaz F, Iftikhar S. Grooming future physician-scientists: evaluating the impact of research motivations, practices, and perceived barriers towards the uptake of an academic career among medical students. Cureus. 2017;9(12): e1991. 29503785 10.7759/cureus.1991 PMC5828671

[11] Mulvale G, Embrett M, Razavi SD. “Gearing Up” to improve interprofessional collaboration in primary care: a systematic review and conceptual framework. BMC Fam Pract. 2016;17:83. 27440181 10.1186/s12875-016-0492-1 PMC4955241

[12] Smith T, McNeil K, Mitchell R, Boyle B, Ries N. A study of macro-, meso- and micro-barriers and enablers affecting extended scopes of practice: the case of rural nurse practitioners in Australia. BMC Nurs. 2019;18(1):14. 30976197 10.1186/s12912-019-0337-z PMC6444450

[13] Desai D, Mayne, C., Bates, H., & Martin, P. A rapid review of barriers and enablers of medical student participation in research in health settings. Open Science Framework 2022; 10.1007/s40670-024-02156-z PMC11699221 39758477

[14] Garritty C, Gartlehner G, Nussbaumer-Streit B, King VJ, Hamel C, Kamel C, et al. Cochrane Rapid Reviews Methods Group offers evidence-informed guidance to conduct rapid reviews. J Clin Epidemiol. 2021;130:13–22. 33068715 10.1016/j.jclinepi.2020.10.007 PMC7557165

[15] Tricco AC, Straus SE, Ghaffar A, Langlois EV. Rapid reviews for health policy and systems decision-making: more important than ever before. Syst Rev. 2022;11(1):153. 35906637 10.1186/s13643-022-01887-7 PMC9338614

[16] The EndNote Team. EndNote. EndNote X9 ed. Philadelphia, PA: Clarivate Analytics; 2013.

[17] Covidence systematic reviews software, web-based software Melbourne, Australia: Veritas Health Innovation; 2021 [cited 2022 June]. Available from: https://www.covidence.org.

[18] Law M SD, Pollock N, Letts L, Bosch J, Westmorland M Critical review form – quantitative studies. McMaster Evidence Review Synthesis Team; 1998.

[19] Hong QNFS, Bartlett G, Boardman F, Cargo M, Dagenais P, Gagnon MP, Griffiths F, Nicolau B, O’Cathain A, Rousseau MC, Vedel I, Pluye P. The Mixed Methods Appraisal Tool (MMAT) version 2018 for information professionals and researchers. Educ Inf. 2018;24(4):285–91.

[20] Hsieh HF, Shannon SE. Three approaches to qualitative content analysis. Qual Health Res. 2005;15(9):1277–88. 16204405 10.1177/1049732305276687

[21] Alamri Y. Factors influencing decisions to become involved in research: a study of pre-clinical medical students from New Zealand. Med Sci Educ. 2019;29(2):489–92. 34457505 10.1007/s40670-019-00717-1 PMC8368863

[22] Alamri Y, Currie W, Magner K, Al-Busaidi IS, Wilkinson TJ, Beckert L. Publication rates of, and attitudes toward, summer research projects: 10-year experience from a single institution in New Zealand. Adv Med Educ Pract. 2019;10:263–71. 31118864 10.2147/AMEP.S198789 PMC6504710

[23] Baig SA, Hasan SA, Ahmed SM, Ejaz K, Aziz S, Dohadhwala NA. Reasons behind the increase in research activities among medical students of Karachi, Pakistan, a low-income country. Educ Health (Abingdon). 2013;26(2):117–21. 24200734 10.4103/1357-6283.120705

[24] Jimmy R, Palatty PL, D’Silva P, Baliga MS, Singh A. Are medical students inclined to do research? J Clin Diagn Res. 2013;7(12):2892–5. 24551667 10.7860/JCDR/2013/6698.3786 PMC3919354

[25] Motwani D, Hada V, Taranikanti M. Research at undergraduate level: medical student’s perspective. 2021.

[26] Omprakash A, Prabu Kumar A, Ramaswamy P, Sathiyasekaran BWC, Ravinder T. Assessment of knowledge, attitude, perceived barriers towards research among first year undergraduate medical students: a study from Chennai, Tamil Nadu, India. J Clin Diagn Res. 2019;13. 10.7860/JCDR/2019/42162.13270

[27] Stockfelt M, Karlsson L, Finizia C. Research interest and activity among medical students in Gothenburg, Sweden, a cross-sectional study. BMC Med Educ. 2016;16(1):226. 27565878 10.1186/s12909-016-0749-3 PMC5002212

[28] Bonilla-Escobar FJ, Bonilla-Velez J, Tobón-García D, Ángel-Isaza AM. Medical student researchers in Colombia and associated factors with publication: a cross-sectional study. BMC Med Educ. 2017;17(1):254. 29246229 10.1186/s12909-017-1087-9 PMC5732498

[29] Kini S, Muthukumar R, Maiya G, Kodyalamoole NK, Kiran N. Attitudes and perceptions towards research among final year medical students in a private medical college of coastal Karnataka: a cross sectional study. Journal of Health and Allied Sciences NU. 2017;07(1):007–11.

[30] Chellaiyan VG, Manoharan A, Jasmine M, Liaquathali F. Medical research: perception and barriers to its practice among medical school students of Chennai. J Educ Health Promot. 2019;8:134. 31463319 10.4103/jehp.jehp_464_18 PMC6691744

[31] Kharraz R, Hamadah R, AlFawaz D, Attasi J, Obeidat AS, Alkattan W, et al. Perceived barriers towards participation in undergraduate research activities among medical students at Alfaisal University-College of Medicine: a Saudi Arabian perspective. Med Teach. 2016;38(Suppl 1):S12–8. 26984028 10.3109/0142159X.2016.1142507

[32] Kumar J, Memon A, Kumar A, Kumari R, Kumar B, Fareed S. Barriers experienced by medical students in conducting research at undergraduate level. Cureus. 2019;11(4): e4452. 31205838 10.7759/cureus.4452 PMC6561510

[33] Nel D, Burman RJ, Hoffman R, Randera-Rees S. The attitudes of medical students to research. S Afr Med J. 2013;104(1):33–6. 24388084 10.7196/samj.7058

[34] Rani R, Priya M. Knowledge, attitude and practice on medical research: the perspective of medical students. Biosci, Biotechnol Res Asia. 2014;11:115–9.

[35] Sayedalamin Z, Halawa TF, Baig M, Almutairi O, Allam H, Jameel T, et al. Undergraduate medical research in the Gulf Cooperation Council (GCC) countries: a descriptive study of the students’ perspective. BMC Res Notes. 2018;11(1):283. 29739473 10.1186/s13104-018-3381-y PMC5941694

[36] Awofeso OM, Roberts AA, Okonkwor CO, Nwachukwu CE, Onyeodi I, Lawal IM, et al. Factors affecting undergraduates’ participation in medical research in Lagos. Niger Med J. 2020;61(3):156–62. 33100468 10.4103/nmj.NMJ_94_19 PMC7547749

[37] Noorelahi MM, Soubhanneyaz AA, Kasim KA. Perceptions, barriers, and practices of medical research among students at Taibah College of Medicine, Madinah. Saudi Arabia Adv Med Educ Pract. 2015;6:479–85. 26185479 10.2147/AMEP.S83978 PMC4500619

[38] Amgad M, Tsui MMK, Liptrott SJ, Shash E. Medical student research: an integrated mixed-methods systematic review and meta-analysis. PLoS One. 2015;10(6):e0127470. 26086391 10.1371/journal.pone.0127470 PMC4472353

[39] Kelley K, Clark B, Brown V, Sitzia J. Good practice in the conduct and reporting of survey research. Int J Qual Health Care. 2003;15(3):261–6. 12803354 10.1093/intqhc/mzg031

