# Supplementary material for: A Review of the Enablers and Barriers of Medical Student Participation in Research
Source: Med Sci Educ. 2024 Sep 4;34(6):1629–39. doi: 10.1007/s40670-024-02156-z (PMC11699221; doi:10.1007/s40670-024-02156-z)
Supplement: Supplementary file 1 — Supplementary file1 (DOCX 40 KB) [file 40670_2024_2156_MOESM1_ESM.docx]

**Supplementary Information**

**Article title:** A review of the enablers and barriers of medical student participation in research

**Journal name:** Medical Science Educator

**Author names:** Chance Mayne, Hannah Bates, Devang Desai & Priya Martin

Affiliation and e-mail address of the corresponding author: The University of Queensland, [Priya.Martin@uq.edu.au](mailto:Priya.Martin@uq.edu.au)

**Supplementary Table 1: WHO Rapid Review Checklist**

| **Category** | **Items to consider** | **Response** |
| --- | --- | --- |
| Protocol | Was a protocol used? | Yes |
|  | If so, was the protocol made public, published in a journal, and/or registered (if so, provide reference and/or registration number, or link to protocol)? | Yes, it was registered on Open Science Framework – <https://doi.org/10.17605/OSF.IO/5XZWN>  It was also uploaded on the ResearchGate webpage of all the authors |
| Overall scope | Was the scope limited in any way? | No |
|  | Were there a limited number of research or policy questions? | No |
|  | Were the research questions of limited type (e.g. effectiveness only, specific populations)? | No |
|  | Was the number of included studies limited? | No |
| Comprehensiveness | Was the search strategy limited in any way (e.g. number of databases, grey literature, date, setting, language)? | The only limitation placed was on the number of databases searched (n=3). |
|  | Were there limits on the types of study designs included (e.g. existing systematic reviews, randomized controlled trials)? | All primary research designs were included. |
|  | Was textual analysis limited (e.g. no full-text review and/or limits on the number of items extracted)? | No |
| Rigour and quality control | Was the process of dual study selection or dual data extraction modified or omitted? | No |
|  | Was the internal or external review of the final research limited or omitted? | No |
| Synthesis | Was the assessment of risk of bias or quality of evidence limited or omitted? | No |
|  | Was qualitative or quantitative analysis limited or omitted? | No |
| Other | When making statements about the findings of the rapid review, were the conclusions simplified or omitted? | No |
|  | Is it appropriate to provide a disclaimer and/or limitations section in context with your findings? | Yes. Limitations of the review have been outlined. |

**Supplementary Table 2: Search strategy for all databases**

1. **PubMed Search Strategy**

| **Search number** | **PubMed Search String** | **Results** |
| --- | --- | --- |
| 1 | "students, medical"[MeSH Terms] | 40,726 |
| 2 | "medical student*"[Title/Abstract] | 48,901 |
| 3 | #1 OR #2 | 64,425 |
| 4 | "Attitude"[MeSH Terms:noexp] | 51,928 |
| 5 | "enabler*"[Title/Abstract] OR "barrier*"[Title/Abstract] OR "opportunity"[Title/Abstract] OR "obstacles"[Title/Abstract] OR "motivat*"[Title/Abstract] OR "outcomes"[Title/Abstract] OR "experience"[Title/Abstract] OR "perception*"[Title/Abstract] OR "engage*"[Title/Abstract] OR "predictors"[Title/Abstract] | 3,074,717 |
| 6 | #4 OR #5 | 3,111,283 |
| 7 | "Biomedical Research"[MeSH Terms:noexp] | 77,908 |
| 8 | "clinical research"[Title/Abstract] OR "medical research"[Title/Abstract] OR "hospital research"[Title/Abstract] OR "extracurricular research"[Title/Abstract] OR "extra-curricular research"[Title/Abstract] OR "extracurricular participation"[Title/Abstract] OR "extra-curricular participation"[Title/Abstract] OR "voluntary research"[Title/Abstract] OR "research participat*"[Title/Abstract] OR "student research"[Title/Abstract] | 86,602 |
| 9 | #7 OR #8 | 151,325 |
| 10 | #3 AND #6 AND #9 | 471 |
| 11 | Limit to: 2012-2022, English language | 308 |

1. **EMBASE Search Strategy**

| **Search Number** | **EMBASE Search string (limit ‘Source’ to EMBASE and Preprints ed)** | **Results** |
| --- | --- | --- |
| 1 | 'medical student'/mj | 31,915 |
| 2 | (medic* NEAR/3 student*):ab,ti | 74,494 |
| 3 | #1 OR #2 (limited to EMBASE OR Preprint) | 54,628 |
| 4 | 'medical research'/mj (limited to EMBASE OR Preprint) | 54,891 |
| 5 | ‘clinical research’/exp(limited to EMBASE OR Preprint) | 85,019 |
| 6 | 'health research*':ab,ti OR 'medical research*':ab,ti OR 'clinical research*':ab,ti OR 'hospital research*':ab,ti OR 'extra-curricular research*':ab,ti OR 'extracurricular research*':ab,ti OR 'extracurricular participat*':ab,ti OR 'extra-curricular participat*':ab,ti OR 'research participat*':ab,ti OR 'student research':ab,ti OR 'voluntary research*':ab,ti | 125,312 |
| 7 | #4 OR #5 OR #6 | 230,748 |
| 8 | enabler*:ab,ti OR barrier*:ab,ti OR opportunit*:ab,ti OR obstacle*:ab,ti OR motivati*:ab,ti OR outcome*:ab,ti OR experience*:ab,ti OR attitude*:ab,ti OR perception*:ab,ti OR predict*:ab,ti OR engage*:ab,ti | 7,493,129 |
| 9 | #3 AND #7 AND #8 | 644 |
| 10 | Limited to: 2012-2022; EMBASE OR Preprint; Document type limited to Article, article in press or review | 173 |

1. **Psycinfo Search Strategy**

| **Search number** | **Psycinfo Search String** | **Results** |
| --- | --- | --- |
| #1 | exp Medical Students/ | 10528 |
| #2 | "medical student*".ab,ti. | 9523 |
| #3 | #1 or #2 | 13299 |
| #4 | (enabler* or barrier* or opportunit* or obstacles or motivat* or outcome* or experience or perception* or engage* or predictor* or prediction*).ab,ti. | 1054488 |
| #5 | *Experimentation/ | 24780 |
| #6 | (clinical research or medical research or hospital research or extracurricular research or extra-curricular research or extracurricular participation or extra-curricular participation or voluntary research or research participation or student research).ab,ti. | 10799 |
| #7 | #5 OR #6 | 34509 |
| #8 | #3 AND #4 AND #7 | 77 |
| #9 | Limit to: 2012-2022 (no option to limit to English) | 40 |

**Supplementary Table 3: Data Extraction Template**

| Author | Country | Setting | Participants | Study design/ Measurement tool | Outcome measures | Barriers | Enablers/ motivators | Overall findings | Limitations/ Future directions |
| --- | --- | --- | --- | --- | --- | --- | --- | --- | --- |
|  |  |  |  |  |  |  |  |  |  |

**Supplementary Table 4: Excluded studies with reasons**

| **Reason for exclusion** | **Reference** |
| --- | --- |
| **Wrong population** (not medical students and studies with medical students involved alongside other professions, but results unable to be separated out for medical students) | Al-Halabi et al. 2014  Ashrafi-Rizi et al. 2015  Steadman et al. 2015  Truncali et al. 2021  Zafar et al. 2021 |
| **Wrong investigated phenomena** (Student perspectives only on research, no actual research involved):  **Wrong intervention**  **Wrong outcome** | Althubaiti 2015  Barron et al. 2015  Bath et al. 2018  Ha et al. 2018  Howell 2021  Abulaban et al. 2017  AlSayegh et al. 2020  Ommering et al. 2021 |
|  |  |
| **Wrong context** (setting other than medical school and healthcare settings) | Hoh et al. 2019  Moller et al. 2015  Nguyen et al. 2020 |
| **Wrong study design** (reviews, editorials and descriptive papers) | Chamberlain et al. 2014  Eissues et al. 2015  Havnaer et al. 2017  Mabvuure et al. 2012  Modell et al. 2014  Yee et al. 2016  Zier et al. 2012 |

**Supplementary Table 5a: Critical Appraisal of Qualitative Studies**

McMaster Quantitative Critical Appraisal tool

Appraisal Questions

- Q1: was the purpose clearly stated?
- Q2: was the relevant literature reviewed?
- Q3: what was the study design?
- Q4: was the sample size described in detail?
- Q5: was the sample size justified?
- Q6: where the outcome measures reliable?
- Q7: where the outcome measures valid?
- Q8: was the intervention described in detail?
- Q9: was contamination avoided?
- Q10: was co-intervention avoided?
- Q11: were results reported in terms of statistical significance?
- Q12: were the analysis methods appropriate?
- Q13: was clinical importance reported?
- Q14: were drop-outs recorded?
- Q15: were the conclusions given appropriate for study methods and results?

| **Study** | **Q1** | **Q2** | **Q3** | **Q4** | **Q5** | **Q6** | **Q7** | **Q8** | **Q9** | **Q10** | **Q11** | **Q12** | **Q13** | **Q14** | **Q15** |
| --- | --- | --- | --- | --- | --- | --- | --- | --- | --- | --- | --- | --- | --- | --- | --- |
| Alamri et al 2019 (b) | Y | Y | CS (Cross-sectional) | Y | N | N | N | N/A | N/A | N/A | Y | Y | Y | Y | Y |
| Alamri et al 2021 | Y | Y | CS | Y | Y | N | N | N/A | N/A | N/A | Y | Y | Y | N | Y |
| AlGhamdi et al 2014 | Y | Y | CS | Y | N | N | N | N/A | N/A | N/A | N | N | Y | N | N |
| Assar et al 2018 | Y | Y | CS | Y | Y | N | Y | N/A | N/A | N/A | Y | Y | Y | N | Y |
| Awofeso et al 2020 | Y | Y | CS | Y | N | N | N | N/A | N/A | N/A | Y | Y | Y | Y | Y |
| Baig et al 2013 | Y | Y | CS | Y | Y | N | N | N/A | N/A | N/A | Y | Y | Y | N | Y |
| Bonilla-Escobar et al 2017 | Y | Y | CS | Y | Y | N | Y | N/A | N/A | N/A | Y | Y | Y | N | Y |
| Chellaiyan et al 2019 | Y | Y | CS | Y | Y | N | N | N/A | N/A | N/A | Y | Y | Y | N | Y |
| Funston et al 2016 | Y | Y | CS | Y | Y | N | N | N/A | N/A | N/A | Y | Y | Y | N | Y |
| Hada et al 2021 | Y | Y | CS | Y | Y | N | Y | N/A | N/A | N/A | N | Y | Y | N | N |
| Rani & Priya 2014 | Y | Y | CS | Y | Y | N | Y | N/A | N/A | N/A | N | Y | Y | N | Y |
| Jimmey et al 2013 | Y | Y | CS | Y | Y | N | Y | N/A | N/A | N/A | N | Y | Y | N | Y |
| Kharraz et al 2016 | Y | Y | CS | Y | N | N | N | N/A | N/A | N/A | N | Y | Y | N | Y |
| Kini et al 2017 | Y | Y | CS | Y | N | N | N | N/A | N/A | N/A | Y | Y | Y | N | Y |
| Kumar et al 2019 | Y | Y | CS | Y | N | N | N | N/A | N/A | N/A | Y | Y | Y | N | Y |
| Mahmood Shah et al 2017 | Y | Y | CS | Y | Y | N | N | N/A | N/A | N/A | Y | Y | Y | Y | Y |
| Muhandiramge et al 2021 | Y | Y | CS | Y | Y | N | N | Y | Y | Y | Y | Y | Y | Y | Y |
| Nel et al 2014 | Y | Y | CS | Y | Y | N | N | N/A | N/A | N/A | Y | Y | Y | N | Y |
| Noorelahi et al 2015 | Y | Y | CS | Y | N | N | N | N/A | N/A | N/A | Y | Y | Y | Y | Y |
| Omprahash et al 2019 | Y | Y | CS | Y | Y | N | N | N/A | N/A | N/A | Y | Y | Y | Y | Y |
| Sayedalamin et al 2018 | Y | Y | CS | Y | Y | N | N | N/A | N/A | N/A | Y | Y | Y | Y | Y |
| Shahab et al 2013 | Y | Y | CS | Y | N | N | N | N/A | N/A | N/A | Y | Y | Y | Y | Y |
| Singh et al 2021 | Y | Y | CS | Y | N | N | Y | N/A | N/A | N/A | Y | Y | Y | Y | Y |
| Sobczuk et al 2022 | Y | Y | CS | Y | Y | N | N | N/A | N/A | N/A | Y | Y | Y | N | Y |
| Stockfelt et al 2016 | Y | Y | CS | Y | N | N | N | N/A | N/A | N/A | Y | Y | Y | N | Y |
| Yerpude and Jogdand 2016[40] | Y | Y | CS | Y | Y | N | N | N/A | N/A | N/A | Y? | Y | Y | N | Y |

**Supplementary Table 5b: Critical Appraisal of the Mixed Methods study**

McGill Mixed Methods Appraisal Tool

1. Is there an adequate rationale for using a mixed methods design to address the research question?
2. Are the different components of the study effectively integrated to answer the research question?
3. Are the outputs of the integration of qualitative and quantitative components adequately interpreted?
4. Are divergences and inconsistencies between quantitative and qualitative results adequately addressed?
5. Do the different components of the study adhere to the quality criteria of each tradition of the methods involved?

|  | **Q1** | **Q2** | **Q3** | **Q4** | **Q5** |
| --- | --- | --- | --- | --- | --- |
| Alamri et al. 2019 (a) | Y | Y | Y | Y | N |

NOTE for Q5: qualitative component when assessed did not adequately address Q1.2
